# Evidence of positive selection at codon sites localized in extracellular domains of mammalian CC motif chemokine receptor proteins

**DOI:** 10.1186/1471-2148-10-139

**Published:** 2010-05-10

**Authors:** Kelsey J Metzger, Michael A Thomas

**Affiliations:** 1 Department of Biological Sciences, Idaho State University, 921 South 8th Avenue, Campus Box 8007, Pocatello, ID 83209, USA; 2 Center for Learning Innovation, University of Minnesota Rochester, 300 University Square, 111 South Broadway, Rochester, MN 55904, USA

## Abstract

**Background:**

CC chemokine receptor proteins (CCR1 through CCR10) are seven-transmembrane G-protein coupled receptors whose signaling pathways are known for their important roles coordinating immune system responses through targeted trafficking of white blood cells. In addition, some of these receptors have been identified as fusion proteins for viral pathogens: for example, HIV-1 strains utilize CCR5, CCR2 and CCR3 proteins to obtain cellular entry in humans. The extracellular domains of these receptor proteins are involved in ligand-binding specificity as well as pathogen recognition interactions.

In mammals, the majority of chemokine receptor genes are clustered together; in humans, seven of the ten genes are clustered in the 3p21-24 chromosome region. Gene conversion events, or exchange of DNA sequence between genes, have been reported in chemokine receptor paralogs in various mammalian lineages, especially between the cytogenetically closely located pairs CCR2/5 and CCR1/3. Datasets of mammalian orthologs for each gene were analyzed separately to minimize the potential confounding impact of analyzing highly similar sequences resulting from gene conversion events.

Molecular evolution approaches and the software package Phylogenetic Analyses by Maximum Likelihood (PAML) were utilized to investigate the signature of selection that has acted on the mammalian CC chemokine receptor (*CCR*) gene family. The results of neutral vs. adaptive evolution (positive selection) hypothesis testing using Site Models are reported. In general, positive selection is defined by a ratio of nonsynonymous/synonymous nucleotide changes (dN/dS, or ω) >1.

**Results:**

Of the ten mammalian CC motif chemokine receptor sequence datasets analyzed, only *CCR2 *and *CCR3 *contain amino acid codon sites that exhibit evidence of positive selection using site based hypothesis testing in PAML. Nineteen of the twenty codon sites putatively indentified as likely to be under positive selection code for amino acid residues located in extracellular domains of the receptor protein products.

**Conclusions:**

These results suggest that amino acid residues present in intracellular and membrane-bound domains are more selectively constrained for functional signal transduction and homo- or heterodimerization, whereas amino acid residues in extracellular domains of these receptor proteins evolve more quickly, perhaps due to heightened selective pressure resulting from ligand-binding and pathogen interactions of extracellular domains.

## Background

Chemotactic or chemoattractant cytokine (chemokine) proteins are a unique division of cytokines characterized by their roles in cell signaling through the use of heterotrimeric GTP-binding (G protein-coupled) 7-transmembrane receptors [1-3]. Chemokines are the largest family of cytokines [4]. Currently, 42 ligand molecules and 19 receptors belong to the chemokine superfamily of cytokines [5]. Within the chemokine superfamily, protein sub-families are distinguished by differences in amino acid sequence motif of four conserved cysteines residues [6]. Chemokine ligands in the CC (β) sub-family have two adjacent cysteines that are both involved in intra-chain disulfide bridges [7]. There are ten CC motif chemokine receptors (CCR1 through CCR10) that bind these ligands with differing ligand binding specificity [2,5].

Chemokines, in conjunction with adhesion molecules, recruit specific subpopulations of leukocytes by activating various receptors and initiating signaling through G protein-coupled pathways [8,9]. The signaling of chemokines through their receptors affect various cellular outcomes including leukocyte trafficking, gene transcription, degranulation of immune response cells, mitogenic processes, and apoptosis [2,5,6,8]. Chemokines generally act as secondary pro-inflammatory mediators that are induced by primary pro-inflammatory mediators [1].

Nucleotide mutations in the open reading frame coding for chemokine receptors can have a dramatic effect on receptor activity or little effect, depending on the location of the substitution and the nature of the amino acid replacement: amino acid substitutions resulting in alterations at key ligand binding extracellular domains or intracellular G-protein coupled domains in particular are known to result in disrupted or abnormal receptor activity [4]. Deficient signal transduction can result in increased susceptibility to infectious diseases as a result of the lack of a robust signaling response to pathogenic infection [10-16]. Mutations in the regulatory nucleotide sequence of chemokine receptors can also result in changes in gene expression and subsequent protein activity [17,18].

In addition to their roles as pro-inflammatory agents in innate immune response through binding of endogenous ligands and subsequent receptor activation, some chemokine receptors have been co-opted to act as receptors or fusion proteins for a number of pathogens including HIV-1 strains [19,20], protozoan parasites *Plasmodium knowlesi *and *P. vivax *[21], and Epstein-Barr virus [22]; herpesviruses mimic host chemokine receptors to elude host immune responses [23]. Mutations in regulatory or coding sequences for chemokine receptors that are used as pathogen fusion proteins can alter host-pathogen interactions: amino acid substitutions in extracellular domains can prevent recognition by the pathogen or interfere with the pathogen's ability to utilize the receptor as a gateway into the cell. Genetic markers associated with disease resistance have been found in regulatory and coding sequences of chemokine receptors [24-29].

Because of their critical role in signaling immune responses, chemokine receptors are subjects of intense selection to accommodate signaling molecules, and are expected to experience purifying selection to maintain conformation and functionality of ligand binding and signaling [30]. However, because of their role as targets of pathogen entry, chemokine receptors are also expected to experience positive selection pressure in response to viral/pathogen hijacking [31]. Loci that are involved in responses to a variety of pathogens may experience balancing selection as a result of diverging selection pressures acting simultaneously on genes which may result in the maintenance of polymorphism [32]. Given this apparent evolutionary tension, it is of interest to investigate the signature of selection on this subfamily of chemokine receptors.

## Results

### Hypothesis Testing with Site Models

This paper presents the results of PAML hypothesis testing on chemokine receptor sequence datasets with "Site" models only [33,34]. These models analyze sequence data at the level of the codon, and test whether a hypothesis (model) that allows for positive selection (dN/dS > 1 for some codons) is better fit to the data when compared to a null neutral hypothesis (model), determined through performing a likelihood ratio test between the likelihood scores of the null neutral and selection models. Each set of orthologous gene sequences was analyzed independently of one another such that only *CCR1 *gene sequences were included in the first data set, only *CCR2 *gene sequences were included in the second data set, and so on for each of the ten CCR genes (i.e., paralogous genes were not in the same data set).

**Figure 1:**
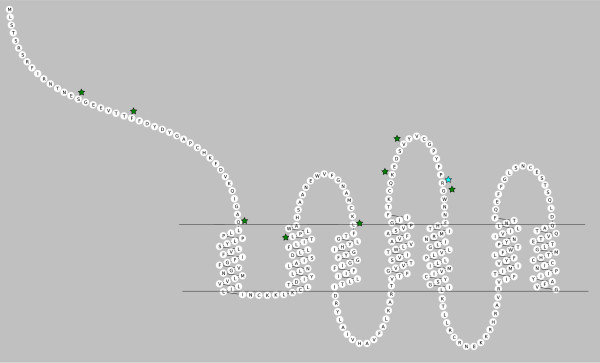
**Location of CCR2 Positively Selected Codon Sites in Extracellular Receptor Protein Domains**. Stars indicate the location of positively selected sites in CCR2: Green stars indicate positively selected sites with a Bayes Empirical Bayes posterior probability ≤ 95%, blue stars indicate positively selected sites with posterior probability ≥95%. Diagram created with RbDe online software application [62].

When testing the hypothesis that some codon sites within chemokine receptor coding sequences have experienced positive selection pressure, significant results were obtained for some codons within the genes CCR2 and CCR3 (Table 1). For CCR2, the comparison between a null neutral site model which does not allow positive selection (M1a) and a selection site model (M2a) yielded a likelihood ratio test statistic of 5.45, which did not allow for rejection of the null hypothesis of neutral selection. However, the comparison between an additional pair of site models, M7 (null, neutral) and M8 (selection) for CCR2 yielded a likelihood test ratio statistic of 15.22, significant at p = 0.001, and a proportion of sites (0.02430, 2.4%) with ω = 2.83526. The analysis for *CCR2 *had a total of 380 amino acid sites, and nine amino acid sites were identified as sites of positive selection (Table 2) using Bayes empirical Bayes (BEB) analysis [35]. One of the nine amino acid sites identified under positive selection had strong support with BEB posterior probability >95%. Eight of the nine amino acids identified as having experienced positive selection in the coding sequence of *CCR2 *are located in extracellular domains of the protein; one positively selected amino acid residue is located in the second transmembrane domain, near the transmembrane/extracellular boundary (Figure 1).

**Table 1:** Model Parameter Estimates, dN/dS Ratios, Log Likelihood Values and Test Statistics for PAML Site Models.

Gene	Model	Parameters	*dN/dS*	*p*	*l*	*2Δl*
*CCR2*	M7: Neutral, beta	p = 0.33949, q = 1.05276	0.2417	2	-4931.71	
	M8: Selection, beta + ω	p_0 _= 0.97570, p = 0.45144	0.2728	4	-4924.10	M7 vs. M8: 15.22***
		q = 1.67059, (p_1 _= 0.02430)				
		ω = 2.83526				
*CCR3*	M1a: Nearly Neutral	ω_0 _= 0.07487, ω_1 _= 1.00	0.4314	2	-5536.21	
		p_0 _= 0.61460, (p_1 _= 0.38540)				
	M2a: Selection	ω_0 _= 0.07422, ω_1 _= 1.00	0.5014	4	-5526.29	M1a vs. M2a: 19.84***
		ω_2 _= 5.44115, p_0 _= 0.60315				
		p_1 _= 0.38339, (p_2 _= 0.01346)				
*CCR3*	M7: Neutral, beta	p = 0.22415, q = 0.35810	0.3850	2	-5534.05	
	M8: Selection, beta + ω	p_0 _= 0.98363, p = 0.23871	0.2662	4	-5522.90	M7 vs. M8: 22.30***
		q = 0.3298, (p_1 _= 0.01637)				
		ω = 4.50735				

The test statistic *2Δl *is compared to a χ^2 ^distribution with 2 degrees of freedom, critical values 5.99, 9.21, and 13.82 at 5%, 1%, and 0.1% significance, respectively. Significant results are indicated by asterisks. Values for non-significant comparisons are presented in Additional File 1.

**Table 2:** Positively Selected Sites Under Different PAML Site Models Using Bayes Empirical Bayes Analysis.

Gene	Model	Codon	Domain	Amino Acid	Posterior Probability	Post Mean +- SE for ω
*CCR2*	M8: Selection, beta+ ω	16	EC	S	0.632	1.536 +- 0.783
		23	EC	F	0.795	1.796 +- 0.781
		43	EC	Q	0.0916	1.977 +- 0.729
		95	MB	L	0.795	1.807 +- 0.810
		115	EC	L	0.623	1.535 +- 0.825
		183	EC	K	0.0926	1.986 +- 0.720
		187	EC	V	0.705	1.678 +- 0.831
		196	EC	R	0.961*	2.028 +- 0.691
		197	EC	G	0.862	1.910 +- 0.771
*CCR3*	M2a: Selection	4	EC	S	0.638	3.459 +- 2.147
		5	EC	L	0.988*	4.851 +- 1.496
		20	EC	V	0.998**	4.881 +- 1.453
		179	EC	T	0.919	4.581+- 1.731
		181	EC	T	0.878	4.378 +- 1.807
*CCR3*	M8: Selection, beta+ ω	4	EC	S	0.824	2.296 +- 0.904
		5	EC	L	0.988*	2.590 +- 0.683
		20	EC	V	0.997**	2.603 +- 0.667
		31	EC	A	0.549	1.734 +- 0.921
		95	EC	R	0.681	2.000 +- 0.947
		96	EC	G	0.598	1.830 +- 0.940
		104	EC	G	0.863	2.347 +- 0.833
		177	EC	L	0.528	1.688 +- 0.902
		179	EC	E	0.550	1.748 +- 0.967
		181	EC	T	0.962*	2.545 +- 0.729

Asterisks indicate posterior probability P > 95% (*) and P > 99%(**). For codon position, the amino acid number is given followed by an abbreviation for the domain in which the amino acid is found: EC = extracellular, MB = membrane bound, IC = intracellular.

**Figure 2:**
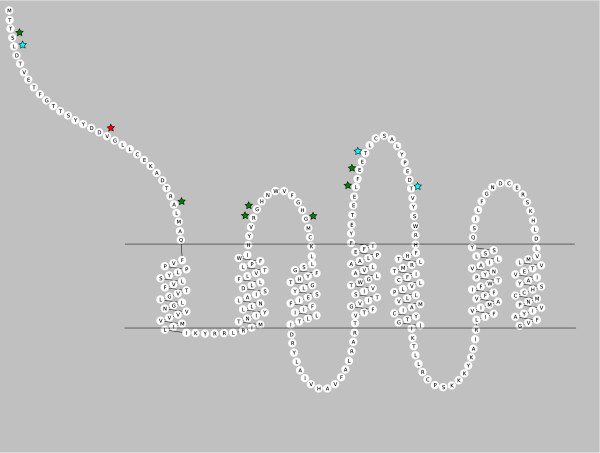
**Location of CCR3 Positively Selected Codon Sites in Extracellular Receptor Protein Domains**. Stars indicate the location of positively selected sites in CCR3: Green stars indicate positively sites with a Bayes Empirical Bayes posterior probability ≤ 95%, blue stars indicate positively sites with posterior probability ≥95%, red stars indicate sites under positive selection with posterior probability of ≥99%. Diagram created with RbDe online software application [62].

For hypothesis testing of site models with CCR3 gene sequences, the comparison of M1a (neutral) vs. M2a (selection) site models led to rejection of the null, neutral hypothesis in favor of selection with a likelihood test ratio statistic of 19.84, significant at p = 0.001, with a proportion of sites (0.01346 1%), and ω = 5.44115, indicating positive selection for those codon sites (Table 1). Five specific codon sites within the coding sequence were reported under positive selection with a BEB analysis following site testing: one site had strong support with BEB posterior probability greater than 95%, and one site had very strong support with BEB posterior probability greater than 99% (Table 2). A significant result was also obtained in the comparison of the second set of site models, M7 versus M8, with a test statistic of 22.3, significant at p = 0.001, with a proportion of sites (0.01637, ~1.6%) having a dN/dS value of 5.44 (Table 1). The analysis for *CCR3 *included 361 amino acid sites, and eleven specific amino acid sites were reported under positive selection with a BEB analysis following the site test. Three of these positively selected amino acid sites had posterior probabilities greater than 95%, one site had a posterior probability greater than 99% (Table 2). All of the positively selected sites identified for *CCR3 *in this analysis were located in extracellular domains of the receptor protein (Figure 2). A summary of results (significant and non significant) for all site model tests is presented in Additional File 1.

## Discussion

None of the ten CC motif chemokine receptors has a signature of positive selection as indicated by an ω value (ratio of nonsynonymous substitutions/synonymous substitutions, dN/dS) greater than one averaged over all codons as determined by hypothesis testing using branch models in PAML (unpublished results). Site tests, which analyze the sequence at the unit of the codon, revealed a proportion of codon sites that display evidence of positive selection (ω > 1) within the coding sequences of *CCR2 *and *CCR3*. The results obtained under the two sets of site models (M1a vs. M2a and M7 vs. M8) differ in some aspects; for example, the more conservative M1a vs. M2a comparison did not reveal statistically significant results for *CCR2 *while the M7 vs. M8 comparison did reveal significant differences, allowing for the identification of positively selected sites. For *CCR3*, while both M1a vs. M2a and M7 vs. M8 were both statistically significant comparisons, the comparison between M7 and M8 identified the same five codon sites that had been identified under M1a vs. M2a comparison as well as additional positively selected sites that were not identified in the M1a vs. M2a comparison. The differences in the results obtained using different models reflect that the M1a vs. M2a comparison is a more conservative test which may fail to detect positively selected sites identified by the less conservative M7 vs. M8 comparison.

It is interesting to note that in the results obtained for *CCR2 *and *CCR3*, nineteen out of the twenty amino acid sites that are identified as having experienced positive selection are located in the extracellular domains of the chemokine receptor proteins, suggesting that nonsynonymous substitutions are occurring, and more often being selected for, in the ligand binding and pathogen interaction regions of the receptors. Previous studies on CCR2, CCR3 and other CC chemokine receptors have identified the amine-terminus and extracellular domains as being important for both the endogenous ligand-binding functions [36-42] as well as for binding efficacy for pathogens in situations where these receptors have been co-opted as fusion proteins [24,43-45]. These previous results coupled with the findings presented here point to the extracellular domains of CC chemokine receptor proteins being especially relevant to studies of the evolution of structure and function of receptors for endogenous ligand binding ability and as targets of pathogen interaction.

Recombination or gene conversion between paralogs in the chemokine receptor family has been investigated and described, particularly for CCR2/5 conversion in several orders of mammals [31,46-50]. Conversion events between CCR1/3 in rodents have also been reported [31]. These conversion events have primarily involved transmembrane regions, but some conversion events have occurred more extensively over *CCR *gene sequences and have impacted extracellular domains; for example the first extracellular loop of *CCR5 *converted by recombination with *CCR2 *in *Mus *[31], extracellular loop 2 of *CCR5 *converted by recombination with *CCR2 *in *Homo *and *Oryctolagus *[31,46,50], and extracellular loop 3 of *CCR3 *converted by recombination with *CCR1 *in *Mus *[31].

Analysis of sequences that have undergone gene conversion can lead to higher rate of false-positives when using maximum likelihood methods to detect positive selection, particularly in small data sets with only a few sequences, although the rate of false positives is only increased moderately [51]. To minimize the impact of gene conversion events on the results of this study and as an alternative to eliminating sequences or parts of sequences that have undergone conversion events, each set of orthologous genes was analyzed independently of one another such that highly similar sequences resulting from gene conversion events within a species involving paralogous genes were not included in the same analysis, but rather were analyzed independently in separate data sets (e.g., only *CCR1 *gene sequences were analyzed together in one data set; *CCR2 *gene sequences were analyzed in a separate data set, and so on). In addition, a Bayes Empirical Bayes (BEB) analysis was used rather than Naïve Empirical Bayes (NEB) to identify putative codons under positive selection as NEB is less conservative and can be more prone to error in smaller data sets [34,35] whereas BEB produces a low rate of false-positives with sequences that have experienced gene conversion [51].

Both of the genes containing positively selected codon sites (*CCR2 *and *CCR3) *have been reported to have undergone gene conversion events as discussed above; however, in the case of *CCR2*, gene conversion events have led to the conversion of *CCR5 *by *CCR2*, whereas in the case of *CCR3*, it is *CCR3 *that has been converted by *CCR1*. The results presented here, in which only one gene of a gene conversion pair displays evidence of positive selection through hypothesis testing, indicate that independent analyses of sequences that have undergone gene conversion may mitigate the detection of false-positives due to gene conversion.

## Conclusions

Site tests, which analyze genetic sequences at the unit of the codon, revealed a proportion of codon sites that display evidence of positive selection (ω > 1) within the coding sequences of mammalian CC motif chemokine receptor genes *CCR2 *and *CCR3*. Nineteen of the twenty amino acid sites identified as having experienced positive selection are located in extracellular domains of the chemokine receptor proteins CCR2 and CCR3. These results suggest that amino acid residues present in intracellular and membrane-bound domains of mammalian CC motif chemokine receptor proteins are more selectively constrained, whereas amino acid residues in extracellular domains of these receptor proteins evolve more quickly, perhaps due to heightened selective pressure resulting from ligand-binding and pathogen interactions of extracellular domains.

## Methods

Genomic coding sequences for CCRs from a number of placental mammals were obtained through searches of the online database NCBI Gene [52]. Taxa included in the data set were chosen using the recently updated placental mammal phylogeny [53] and the online software application TimeTree [54,55] to estimate divergence times between taxa (estimates given for nuclear genes were used). For PAML analyses, taxa that have diverged more than ~100 MYA may lead to decreased analytical power due to highly divergent sequences, difficulty in sequence alignment, and saturation of substitutions [56-58]; therefore, only taxa that have diverged less than 100 MYA were included in the data set.

For genes that displayed alternative splicing patterns, the presumed ancestral isoform sequence was identified through alignment methods and included in the dataset while the alternative isoforms were not. For *CCR2*, the human isoform that localizes to the plasma membrane was included in the CCR2 dataset, while the cytoplasmic variant was not. Species and GenBank accession numbers for sequences used in the analyses are listed in Additional File 2.

Nucleotide alignments of chemokine receptor sequences were generated using amino acid sequence alignments and the software program TranAlign [59]. The output from TranAlign was converted to Nexus/PAUP format and submitted to the software program ModelTest [60] for selection of the most appropriate model of evolution for each dataset by testing the fit of 56 different evolutionary models with the data set. ModelTest uses both hierarchical likelihood ratio testing and Akaike Information Criterion (AIC). The best model was chosen based on AIC score and the number of estimated parameters. If there was a statistically insignificant difference between two models, the model with the fewest number of estimated parameters was chosen to introduce the least amount of uncertainty to the evolutionary analyses. Models used for analyses are summarized in Additional File 3.

### Phylogenetic Analysis Using Parsimony* (PAUP*) and Phylogenetic Analysis by Maximum Likelihood (PAML) Methods

Maximum likelihood phylogenetic trees for each data set (Additional Files 4, 5, 6, 7, 8, 9, 10, 11, 12 and 13) were constructed with the software package PAUP* [61]. Data sets and maximum likelihood phylogenetic trees for each gene were submitted to PAML CODEML version 4.1 under different models and parameters to test for adaptive evolution either at codon sites ("Site Model"), along lineages ("Branch Model"), or at sites within lineages ("Branch-Site Model") [34]. This paper presents the results of testing the data sets described with "Site" models only.

"Site" models allow the dN/dS ratio to vary across codons within a sequence for a lineage. Proportions of sites within each lineage were estimated to be in different categories: positive selection is indicated by some codons having a dN/dS > 1. The null, neutral model does not allow positive selection and is compared to the alternative hypothesis in which in which positive selection is allowed.

Two sets of site models are commonly used to test hypotheses of selection, and have been used here: M1a vs. M2a and M7 vs. M8. In the first set of models, the model M1a: Nearly Neutral allows 2 categories of codon sites in *p*_0_, and *p*_1 _proportions, with ω_0 _< 1, and ω_1 _= 1, whereas the model M2a: Selection allows an additional category of codons (*p*_2_) with ω_2 _> 1, indicating positive selection. The second set of site models compared is M7 and M8, in which M7 specifies a neutral model with dN/dS ratios across a continuous beta distribution with estimated parameters p and q of the beta distribution, and M8 specifies a similar model with an additional category for sites that have dN/dS > 1, indicating positive selection. M7 assumes a beta distribution of ω values between 0 and 1, and therefore does not allow any sites under positive selection (ω > 1). The M8 model is similar to M7 in that it also assumes a beta distribution for omega values, but allows another category of sites in which ω > 1. The comparison between M7: beta and M8: beta +ω is less conservative, and may indicate positive selection even when none is detected by the M1a: M2a comparison.

The PAML settings for the null (neutral) model M1a were model = 0, NSsites = 1, and for the alternative (selection) model M2a were model = 0, NSsites = 2. The PAML settings for the null model M7 were model = 0, NSsites = 7, and for the alternative (selection) model M8 were model = 0, NSsites = 8.

The likelihood estimates for each were compared using a hierarchical Likelihood Ratio Test (hLRT) of twice the difference in log likelihood values of the models being compared (2ΔlnL), with the result approximating chi-square distribution with degrees of freedom for the test statistic determined by the difference in estimated parameters between the models being compared. For both the M1a (neutral) vs. M2a (selection) and M7 (beta) vs. M8 (beta + selection) comparisons, the null model has two estimated parameters, while the alternative estimates four, resulting in two degrees of freedom and chi-square critical values of 5.99, 9.21, and 13.82 at 5%, 1%, and 0.1% significance, respectively [34].

## Authors' contributions

KM carried out dataset construction, molecular evolution analyses, and drafting of the manuscript. MT contributed to the conception and design of the study, and participated in critical review and revision of the manuscript. All authors read and approved the final manuscript.

## Supplementary Material

Additional file 1 **Supplementary Table 1: Model Parameter Estimates, dN/dS Ratios, Log Likelihood Values and Test Statistics for PAML Site Models**. Summary of results for all PAML hypothesis testing using site models. Includes significant and non-significant results. Click here for file

Additional file 2 **Supplementary Table 2: Taxa and NCBI GenBank accession numbers for loci included in CC chemokine receptor data sets**. List of species and NCBI GenBank accession numbers for sequences used to construct the ten datasets (for each of the ten sets of orthologous genes) for hypothesis testing. Species and accession numbers for each dataset are grouped together on the table. Click here for file

Additional file 3 **Supplementary Table 3: Parameters for ModelTest models of evolution used in PAML hypothesis testing of CC chemokine receptor sequences**. Summary of evolutionary model parameters used in PAML hypothesis testing. Click here for file

Additional file 4 **Supplementary Figure 1: Maximum likelihood phylogeny of mammalian *CCR1 *gene sequences**. A phylogeny of mammalian *CCR1 *gene sequences. The tree was produced using PHYML with the GTR nucleotide model, a discrete gamma model with four categories, and a shape parameter of 0.6525. Bootstrapping was performed with 100 replicates. Bootstrap support is indicated at nodes. Click here for file

Additional file 5 **Supplementary Figure 2: Maximum likelihood phylogeny of mammalian *CCR2 *gene sequences**. A phylogeny of mammalian *CCR2 *gene sequences. The tree was produced using PHYML with the GTR nucleotide model, a discrete gamma model with four categories, and a shape parameter of 0.5867. Bootstrapping was performed with 100 replicates. Bootstrap support is indicated at nodes. Click here for file

Additional file 6 **Supplementary Figure 3: Maximum likelihood phylogeny of mammalian *CCR3 *gene sequences**. A phylogeny of mammalian *CCR3 *gene sequences. The tree was produced using PHYML with the GTR nucleotide model, a discrete gamma model with four categories, proportion of invariable sites 0.3247 and a shape parameter of 2.8971. Bootstrapping was performed with 100 replicates. Bootstrap support is indicated at nodes. Click here for file

Additional file 7 **Supplementary Figure 4: Maximum likelihood phylogeny of mammalian *CCR4 *gene sequences**. A phylogeny of mammalian *CCR4 *gene sequences. The tree was produced using PHYML with the GTR nucleotide model, a discrete gamma model with four categories, proportion of invariable sites 0.5901 and an estimated shape parameter of 33.284. Bootstrapping was performed with 100 replicates. Bootstrap support is indicated at nodes. Click here for file

Additional file 8 **Supplementary Figure 5: Maximum likelihood phylogeny of mammalian *CCR5 *gene sequences**. A phylogeny of mammalian *CCR5 *gene sequences. The tree was produced using PHYML with the HKY nucleotide model, a discrete gamma model with four categories, and a shape parameter of 0.451. Bootstrapping was performed with 100 replicates. Bootstrap support is indicated at nodes. Click here for file

Additional file 9 **Supplementary Figure 6: Maximum likelihood phylogeny of mammalian *CCR6 *gene sequences**. A phylogeny of mammalian *CCR6 *gene sequences. The tree was produced using PHYML with the JC69 nucleotide model, a discrete gamma model with four categories, proportion of invariable sites 0.4667, and an estimated shape parameter of 100. Bootstrapping was performed with 100 replicates. Bootstrap support is indicated at nodes. Click here for file

Additional file 10 **Supplementary Figure 7: Maximum likelihood phylogeny of mammalian *CCR7 *gene sequences**. A phylogeny of mammalian *CCR7 *gene sequences. The tree was produced using PHYML with the GTR nucleotide model, a discrete gamma model with four categories, and a shape parameter of 0.257. Bootstrapping was performed with 100 replicates. Bootstrap support is indicated at nodes. Click here for file

Additional file 11 **Supplementary Figure 8: Maximum likelihood phylogeny of mammalian *CCR8 *gene sequences**. A phylogeny of mammalian *CCR8 *gene sequences. The tree was produced using PHYML with the HKY nucleotide model, a discrete gamma model with four categories, and a shape parameter of 0.6482. Bootstrapping was performed with 100 replicates. Bootstrap support is indicated at nodes. Click here for file

Additional file 12 **Supplementary Figure 9: Maximum likelihood phylogeny of mammalian *CCR9 *gene sequences**. A phylogeny of mammalian *CCR9 *gene sequences. The tree was produced using PHYML with the HKY nucleotide model, a discrete gamma model with four categories, and a shape parameter of 0.3547. Bootstrapping was performed with 100 replicates. Bootstrap support is indicated at nodes. Click here for file

Additional file 13 **Supplementary Figure 10: Maximum likelihood phylogeny of mammalian *CCR10 *gene sequences**. A phylogeny of mammalian *CCR10 *gene sequences. The tree was produced using PHYML with the GTR nucleotide model, a discrete gamma model with four categories, and a shape parameter of 0.2439. Bootstrapping was performed with 100 replicates. Bootstrap support is indicated at nodes. Click here for file
