# Supplementary material for: Evidence of positive selection at codon sites localized in extracellular domains of mammalian CC motif chemokine receptor proteins
Source: BMC Evol Biol. 2010 May 10;10:139. doi: 10.1186/1471-2148-10-139 (PMC2880985; doi:10.1186/1471-2148-10-139)
Supplement: Additional file 1 — Supplementary Table 1: Model Parameter Estimates, dN/dS Ratios, Log Likelihood Values and Test Statistics for PAML Site Models. Summary of results for all PAML hypothesis testing using site models. Includes significant and non-significant results. [file 1471-2148-10-139-S1.DOCX]

**Supplementary Table 1:**  **Model Parameter Estimates, dN/dS Ratios, Log Likelihood Values and Test Statistics for PAML Site Models**.

| **Gene** | **Model** | **Parameters** | ***dN/dS*** | ***p*** | ***l*** | ***2∆l*** |
| --- | --- | --- | --- | --- | --- | --- |
| *CCR1* | M1a: Nearly Neutral | ω0=0.08122, ω1=1.00 | 0.2936 | 2 | -4966.59 |  |
|  |  | p0=0.76880, (p1=0.23120) |  |  |  |  |
|  | M2a: Selection | ω0=0.05026, ω1=1.00 | 0.2936 | 4 | -4966.59 | M1a vs. M2a: 0 |
|  |  | ω2=1.00, p0=0.80349 |  |  |  |  |
|  |  | p1=0.19651, (p2=0.09587) |  |  |  |  |
| *CCR1* | M7: Neutral, beta | p= 0.27099, q= 0.80865 | 0.2498 | 2 | -4961.57 |  |
|  | M8: Selection, beta + ω | p0= 0.95002, p= 0.34550 | 0.2662 | 4 | -4959.32 | M7 vs. M8: 4.50 |
|  |  | q= 1.35446, (p1=0.04998) |  |  |  |  |
|  |  | ω = 1.52991 |  |  |  |  |
| *CCR2* | M1a: Nearly Neutral | ω0=0.10122, ω1=1.00 | 0.2125 | 2 | -4930.07 |  |
|  |  | p0=0.81560, (p1=0.18440) |  |  |  |  |
|  | M2a: Selection | ω0=0.10445, ω1=1.00 | 0.3012 | 4 | -4927.39 | M1a vs. M2a: 5.36 |
|  |  | ω2=4.62095, p0=0.81593 |  |  |  |  |
|  |  | p1=0.17526, (p2=0.00881) |  |  |  |  |
| *CCR2* | M7: Neutral, beta | p= 0.33949, q= 1.05276 | 0.2417 | 2 | -4931.71 |  |
|  | M8: Selection, beta + ω | p0= 0.97570, p= 0.45144 | 0.2728 | 4 | -4924.10 | M7 vs. M8: **15.22***** |
|  |  | q= 1.67059, (p1=0.02430) |  |  |  |  |
|  |  | ω=2.83526 |  |  |  |  |
| *CCR3* | M1a: Nearly Neutral | ω0=0.07487, ω1=1.00 | 0.4314 | 2 | -5536.21 |  |
|  |  | p0=0.61460, (p1=0.38540) |  |  |  |  |
|  | M2a: Selection | ω0=0.07422, ω1=1.00 | 0.5014 | 4 | -5526.29 | M1a vs. M2a: **19.84***** |
|  |  | ω2=5.44115, p0=0.60315 |  |  |  |  |
|  |  | p1=0.38339, (p2=0.01346) |  |  |  |  |
| *CCR3* | M7: Neutral, beta | p= 0.22415, q= 0.35810 | 0.3850 | 2 | -5534.05 |  |
|  | M8: Selection, beta + ω | p0= 0.98363, p= 0.23871 | 0.2662 | 4 | -5522.90 | M7 vs. M8: **22.30***** |
|  |  | q= 0.3298, (p1=0.01637) |  |  |  |  |
|  |  | ω= 4.50735 |  |  |  |  |
| *CCR4* | M1a: Nearly Neutral | ω0=0.05723, ω1=1.00 | 0.0970 | 2 | -3148.30 |  |
|  |  | p0=0.95780, (p1=0.04220) |  |  |  |  |
|  | M2a: Selection | ω0=0.05723, ω1=1.00 | 0.0970 | 4 | -3148.30 | M1a vs. M2a: 0 |
|  |  | ω2=1.00, p0=0.95780 |  |  |  |  |
|  |  | p1=0.02086, (p2=0.02135) |  |  |  |  |
| *CCR4* | M7: Neutral, beta | p= 0.33358, q= 3.46993 | 0.0825 | 2 | -3142.30 |  |
|  | M8: Selection, beta + ω | p0= 0.99999, p= 0.33361 | 0.0825 | 4 | -3142.30 | M7 vs. M8: 0 |
|  |  | q= 3.47047, (p1=0.00001) |  |  |  |  |
|  |  | ω =1.00 |  |  |  |  |
| *CCR5* | M1a: Nearly Neutral | ω0=0.07055, ω1=1.00 | 0.2774 | 2 | -4494.10 |  |
|  |  | p0=0.77743, (p1=0.22257) |  |  |  |  |
|  | M2a: Selection | ω0=0.07055, ω1=1.00 | 0.2774 | 4 | -4494.10 | M1a vs. M2a: 0 |
|  |  | ω2=1.00, p0=0.77743 |  |  |  |  |
|  |  | p1=0.20270, (p2=0.01987) |  |  |  |  |
| *CCR5* | M7: Neutral, beta | p= 0.22029, q= 0.68867 | 0.2416 | 2 | -4491.55 |  |
|  | M8: Selection, beta + ω | p0= 0.99245, p= 0.23692 | 0.2538 | 4 | -4490.11 | M7 vs. M8: 2.88 |
|  |  | q= 0.77911, (p1= 0.0075) |  |  |  |  |
|  |  | ω = 3.12619 |  |  |  |  |
| *CCR6* | M1a: Nearly Neutral | ω0=0.05026, ω1=1.00 | 0.2369 | 2 | -3975.33 |  |
|  |  | p0=0.80349, (p1=0.19651) |  |  |  |  |
|  | M2a: Selection | ω0=0.05026, ω1=1.00 | 0.2369 | 4 | -3975.33 | M1a vs. M2a: 0 |
|  |  | ω2=44.64716, p0=0.80349 |  |  |  |  |
|  |  | p0=0.80349, (p1=0.19651) |  |  |  |  |
| *CCR6* | M7: Neutral, beta | p= 0.19423, q= 0.82650 | 0.1883 | 2 | -3971.75 |  |
|  | M8: Selection, beta + ω | p0= 0.87815, p= 0.31970 | 0.2011 | 4 | -3970.44 | M7 vs. M8: 2.62 |
|  |  | q= 3.02424, (p1= 0.12185) |  |  |  |  |
|  |  | ω = 1.00 |  |  |  |  |
| *CCR7* | M1a: Nearly Neutral | ω0=0.02652, ω1=1.00 | 0.1077 | 2 | -3457.59 |  |
|  |  | p0=0.91657, (p1=0.08343) |  |  |  |  |
|  | M2a: Selection | ω0=0.02652, ω1=1.00 | 0.1077 | 4 | -3457.59 | M1a vs. M2a: 0 |
|  |  | ω2=1.00, p0=0.91657 |  |  |  |  |
|  |  | p1=0.05290, (p2=0.03053) |  |  |  |  |
| *CCR7* | M7: Neutral, beta | p= 0.13428, q= 1.64792 | 0.0681 | 2 | -3446.09 |  |
|  | M8: Selection, beta + ω | p0= 0.99488, p= 0.13924 | 0.0687 | 4 | -3446.04 | M7 vs. M8: 0.10 |
|  |  | q= 1.81935, (p1= 0.00512) |  |  |  |  |
|  |  | ω = 1.00 |  |  |  |  |
| *CCR8* | M1a: Nearly Neutral | ω0=0.08393, ω1=1.00 | 0.3069 | 2 | -3586.69 |  |
|  |  | p0=0.75659, (p1=0.24341) |  |  |  |  |
|  | M2a: Selection | ω0=0.08393, ω1=1.00 | 0.3069 | 4 | -3586.69 | M1a vs. M2a: 0 |
|  |  | ω2=1.00, p0=0.75659 |  |  |  |  |
|  |  | p1=0.15070, (p2=0.09271) |  |  |  |  |
| *CCR8* | M7: Neutral, beta | p= 0.29433, q= 0.79636 | 0.2688 | 2 | -3586.90 |  |
|  | M8: Selection, beta + ω | p0= 0.81210, p= 0.66953 | 0.2881 | 4 | -3586.52 | M7 vs. M8: 0.76 |
|  |  | q= 4.58150, (p1= 0.18790) |  |  |  |  |
|  |  | ω = 1.00 |  |  |  |  |
| *CCR9* | M1a: Nearly Neutral | ω0=0.06830, ω1=1.00 | 0.1638 | 2 | -3590.85 |  |
|  |  | p0=0.89751, (p1=0.10249) |  |  |  |  |
|  | M2a: Selection | ω0=0.06830, ω1=1.00 | 0.1638 | 4 | -3590.85 | M1a vs. M2a: 0 |
|  |  | ω2=40.49411, p0=0.89751 |  |  |  |  |
|  |  | p1=0.10249, (p2=0.00) |  |  |  |  |
| *CCR9* | M7: Neutral, beta | p= 0.30200, q= 1.93498 | 0.1298 | 2 | -3583.06 |  |
|  | M8: Selection, beta + ω | p0= 0.9999, p= 0.30104 | 0.1298 | 4 | -3583.06 | M7 vs. M8: 0 |
|  |  | q= 1.92842, (p1= 0.00001) |  |  |  |  |
|  |  | ω = 3.36779 |  |  |  |  |
| *CCR10* | M1a: Nearly Neutral | ω0=0.04810, ω1=1.00 | 0.1007 | 2 | -3328.59 |  |
|  |  | p0=0.94478, (p1=0.05522) |  |  |  |  |
|  | M2a: Selection | ω0=0.04810, ω1=1.00 | 0.1007 | 4 | -3328.59 | M1a vs. M2a: 0 |
|  |  | ω2=1.00, p0=0.94478 |  |  |  |  |
|  |  | p1=0.02768, (p2=0.02754) |  |  |  |  |
| *CCR10* | M7: Neutral, beta | p= 0.22077, q= 2.23603 | 0.0837 | 2 | -3324.24 | M7 vs. M8: 0 |
|  | M8: Selection, beta + ω | p0= 0.99999, p= 0.22079 | 0.0837 | 4 | 3324.24 |  |
|  |  | q= 2.23642, (p1= 0.00001) |  |  |  |  |
|  |  | ω = 1.00 |  |  |  |  |

Table legend: Summary results for significant and non-significant hypothesis testing using site models. The test statistic *2Δl* is compared to a χ2 distribution with 2 degrees of freedom, critical values 5.99, 9.21, and 13.82 at 5%, 1%, and 0.1% signiﬁcance, respectively.
