# Supplementary material for: Evidence of positive selection at codon sites localized in extracellular domains of mammalian CC motif chemokine receptor proteins
Source: BMC Evol Biol. 2010 May 10;10:139. doi: 10.1186/1471-2148-10-139 (PMC2880985; doi:10.1186/1471-2148-10-139)
Supplement: Additional file 2 — Supplementary Table 2: Taxa and NCBI GenBank accession numbers for loci included in CC chemokine receptor data sets. List of species and NCBI GenBank accession numbers for sequences used to construct the ten datasets (for each of the ten sets of orthologous genes) for hypothesis testing. Species and accession numbers for each dataset are grouped together on the table. [file 1471-2148-10-139-S2.DOCX]

**Supplementary Table 2: Taxa and NCBI GenBank accession numbers for loci included in CC chemokine receptor data sets.**

##### Species Common Name Accession Number

##### *CCR1*

*Bos taurus* cow (breed: Hereford) NM_001077839.1

*Callithrix jacchus* common marmoset AF127528

*Canis lupus familiaris* dog (breed: Boxer) NM_001038606.1

*Felis catus* cat NM_001135969.1

*Homo sapiens* human NM_001295.2

*Macaca mulatta* rhesus monkey NM_001032858.1

*Mus musculus* field mouse NM_009912.4

*Pan troglodytes* chimp XM_516415.2

*Rattus norvegicus* Norway rat NM_020542.2

*Sus scrofa* pig NM_001001621.1

##### *CCR2*

*Bos taurus* cow (breed: Hereford) XM_584158.3

*Canis lupus familiaris* dog (breed: Boxer) XM_541906.1

*Felis catus* cat NM_001097580.1

*Equus caballus* horse NM_001097606.1

*Homo sapiens* human NM_001123396

*Macaca mulatta* rhesus monkey NM_001032806.1

*Mus musculus* field mouse NM_009915.2

*Pan troglodytes* chimp XM_001149515.1

*Rattus norvegicus* Norway rat NM_021866.1

*Sus scrofa* pig NM_001001619.1

##### *CCR3*

*Bos taurus* cow (breed: Hereford) XM_869148.2

*Canis lupus familiaris*  dog (breed: Boxer) NM_001005261.1

*Equus caballus* horse XM_001496014.1

*Homo sapiens* human NM_001837.3

*Felis catus* cat NM_001135970.1

*Mus musculus*  field mouse NM_009914.4

*Macaca fascicularis* crab-eating macaque AY065647

*Macaca mulatta* rhesus monkey NM_001047140.1

*Ovis aries* sheep NM_001009241.1

*Pan troglodytes* chimp XM_001149237.1

*Rattus norvegicus* Norway rat NM_053958.1

*Sus scrofa* pig NM_001001620.1

##### *CCR4*

*Bos taurus* cow (breed: Hereford) NM_001100293.1

*Canis lupus familiaris* dog (breed: Boxer) NM_001003020.1

*Homo sapiens*  human NM_005508.4

*Macaca mulatta* rhesus monkey XM_001098807.1

*Mus musculus* field mouse NM_009916.2

*Pan troglodytes* chimp XM_001168244.1

*Rattus norvegicus* Norway rat NM_133532.2

##### *CCR5*

*Bos taurus*  cow (breed: Hereford) NM_001011672.2

*Canis lupus familiaris* dog (breed: Boxer) NM_001012342.2

*Equus caballus* horse NM_001091534.1

*Felis catus* cat NM_001009248.1

*Homo sapiens*  human NM_000579.3

*Macaca mulatta* rhesus monkey NM_001042773.2

*Mus musculus* field mouse NM_009917.5

*Pan troglodytes* chimp NM_001009046.1

*Papio anubis* olive baboon NM_001112629.1

*Pongo abelii* Sumatran orangutan NM_001135497.1

*Rattus norvegicus* Norway rat NM_053960.3

*Sus scrofa* pig NM_001001618.1

*CCR6*

*Bos taurus* cow (breed: Hereford) XM_597941.2

*Canis lupus familiaris* dog (breed: Boxer) XM_846017.1

*Homo sapiens*  human NM_031409.3

*Macaca mulatta* rhesus monkey NM_001032935.1

*Mus musculus* field mouse NM_009835.3

*Rattus norvegicus* Norway rat NM_001013145.1

*CCR7*

*Bos taurus* cow (breed: Hereford) NM_001024930.2

*Canis lupus familiaris*  dog (breed: Boxer) XM_548131.2

*Homo sapiens* human NM_001838.2

*Macaca mulatta* rhesus monkey NM_001032884.1

*Mus musculus* field mouse NM_007719.2

*Pan troglodytes* chimp XM_511477.2

*Rattus norvegicus* Norway rat NM_199489.3

*Sus scrofa* pig NM_001001532.2

*CCR8*

*Bos taurus*  cow (breed: Hereford) XM_871109.2

*Canis lupus familiaris*  dog (breed: Boxer) XM_542719.2

##### *Homo sapiens* human NM_005201.3

*Macaca mulatta* rhesus monkey XM_001084047.1

*Mus musculus*  field mouse NM_007720.2

*Pan troglodytes* chimp XM_526178.2

*Rattus norvegicus* Norway rat XM_236704.2

*CCR9*

*Bos taurus* cow (breed: Hereford) NM_001098068.1

*Canis lupus familiaris* dog (breed: Boxer) XM_541909.2

*Homo sapiens*  human NM_031200.1

*Mus musculus*  field mouse NM_009913.5

*Ovis aries* sheep NM_001040286.1

*Pan troglodytes* chimp XM_001148175.1

*Rattus norvegicus* Norway rat NM_172329.1

*Sus scrofa* pig NM_001001624.1

*CCR10*

*Bos taurus* cow (breed: Hereford) XM_584874.4

*Canis lupus familiaris*  dog (breed: Boxer) XM_844228.1

*Homo sapiens* human NM_016602.2

*Macaca mulatta* rhesus monkey XM_001111554.1

*Mus musculus*  field mouse NM_007721.4

*Ovis aries* sheep NM_001040287.1

*Pan troglodytes* chimp XM_001162702.1

*Rattus norvegicus* Norway rat NM_001108836.1

*Sus scrofa* pig NM_001044563.1
