# Supplementary material for: Evidence of positive selection at codon sites localized in extracellular domains of mammalian CC motif chemokine receptor proteins
Source: BMC Evol Biol. 2010 May 10;10:139. doi: 10.1186/1471-2148-10-139 (PMC2880985; doi:10.1186/1471-2148-10-139)
Supplement: Additional file 3 — Supplementary Table 3: Parameters for ModelTest models of evolution used in PAML hypothesis testing of CC chemokine receptor sequences. Summary of evolutionary model parameters used in PAML hypothesis testing. [file 1471-2148-10-139-S3.DOCX]

**Supplementary Table 3: Parameters for ModelTest models of evolution used in PAML hypothesis testing of CC chemokine receptor sequences.**

Gene: *CCR1*

Model selected: GTR+G

PAUP Block: BEGIN PAUP;

Lset Base=(0.2056 0.2889 0.2386) Nst=6 Rmat=(1.7579 7.7961 0.9739 2.0352 5.9203) Rates=gamma Shape=0.6525 Pinvar=0;

END;

Gene: *CCR2*

Model selected: TVM+G

PAUP Block: BEGIN PAUP;

Lset Base=(0.2323 0.2619 0.2344) Nst=6 Rmat=(1.4400 5.4881 0.7480 1.5821 5.4881) Rates=gamma Shape=0.5867 Pinvar=0;

END;

Gene: *CCR3*

Model selected: K81uf+I+G

PAUP Block: BEGIN PAUP;

Lset Base=(0.2248 0.2674 0.2335) Nst=6 Rmat=(1.0000 3.6524 0.6678 0.6678 3.6524) Rates=gamma Shape=2.8971 Pinvar=0.3247;

END;

Gene: *CCR4*

Model selected: GTR+I

PAUP Block: BEGIN PAUP;

Lset Base=(0.2078 0.2913 0.2344) Nst=6 Rmat=(1.4792 5.5523 0.4064 1.9465 7.6054) Rates=equal Pinvar=0.5901;

END;

Gene: *CCR5*

Model selected: HKY+G

PAUP Block: BEGIN PAUP;

Lset Base=(0.2390 0.2815 0.2202) Nst=2 TRatio=2.6531 Rates=gamma Shape=0.4471 Pinvar=0;

END;

Gene: *CCR6*

Model selected: TVM+I

PAUP Block: BEGIN PAUP;

Lset Base=(0.2208 0.2746 0.2505) Nst=6 Rmat=(1.7485 5.4650 0.5068 2.3576 5.4650) Rates=equal Pinvar=0.4670;

END;

Gene: *CCR7*

Model selected: TVM+G

PAUP Block: BEGIN PAUP;

Lset Base=(0.1988 0.3091 0.2644) Nst=6 Rmat=(2.0394 8.9836 0.3780 3.3086 8.9836) Rates=gamma Shape=0.2570 Pinvar=0;

END;

Gene: *CCR8*

Model selected: HKY+G

PAUP Block: BEGIN PAUP;

Lset Base=(0.2257 0.2738 0.2312) Nst=2 TRatio=3.0823 Rates=gamma Shape=0.6423 Pinvar=0;

END;

Gene: *CCR9*

Model selected: HKY+G

PAUP Block: BEGIN PAUP;

Lset Base=(0.2116 0.2898 0.2291) Nst=2 TRatio=3.3543 Rates=gamma Shape=0.3564 Pinvar=0;

END;

Gene: *CCR10*

Model selected: TVM+G

PAUP Block: BEGIN PAUP;

Lset Base=(0.1238 0.3656 0.3104) Nst=6 Rmat=(2.3233 6.1148 1.4285 0.6156 6.1148) Rates=gamma Shape=0.2439 Pinvar=0;

END;
