# Supplementary figures and images for: Evidence of positive selection at codon sites localized in extracellular domains of mammalian CC motif chemokine receptor proteins
Source: BMC Evol Biol. 2010 May 10;10:139. doi: 10.1186/1471-2148-10-139 (PMC2880985; doi:10.1186/1471-2148-10-139)

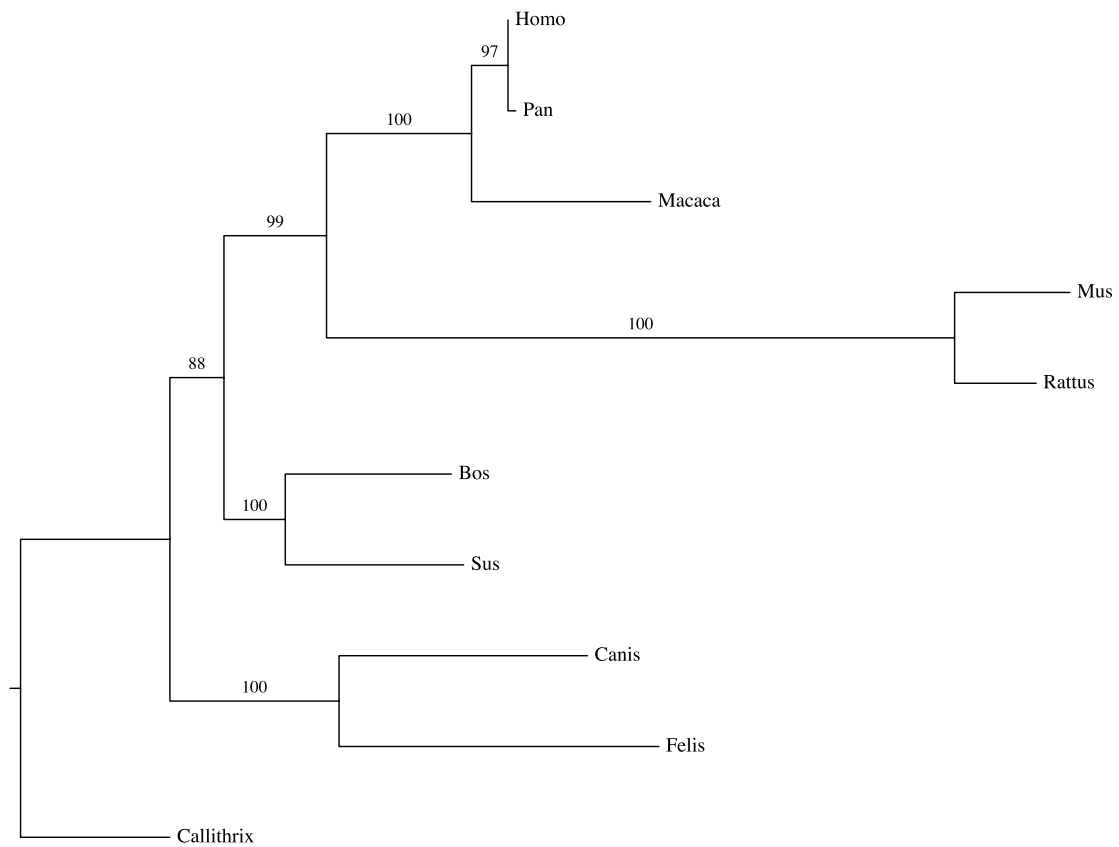

Supplement: Additional file 4 — Supplementary Figure 1: Maximum likelihood phylogeny of mammalian CCR1 gene sequences. A phylogeny of mammalian CCR1 gene sequences. The tree was produced using PHYML with the GTR nucleotide model, a discrete gamma model with four categories, and a shape parameter of 0.6525. Bootstrapping was performed with 100 replicates. Bootstrap support is indicated at nodes. [file 1471-2148-10-139-S4.PDF]

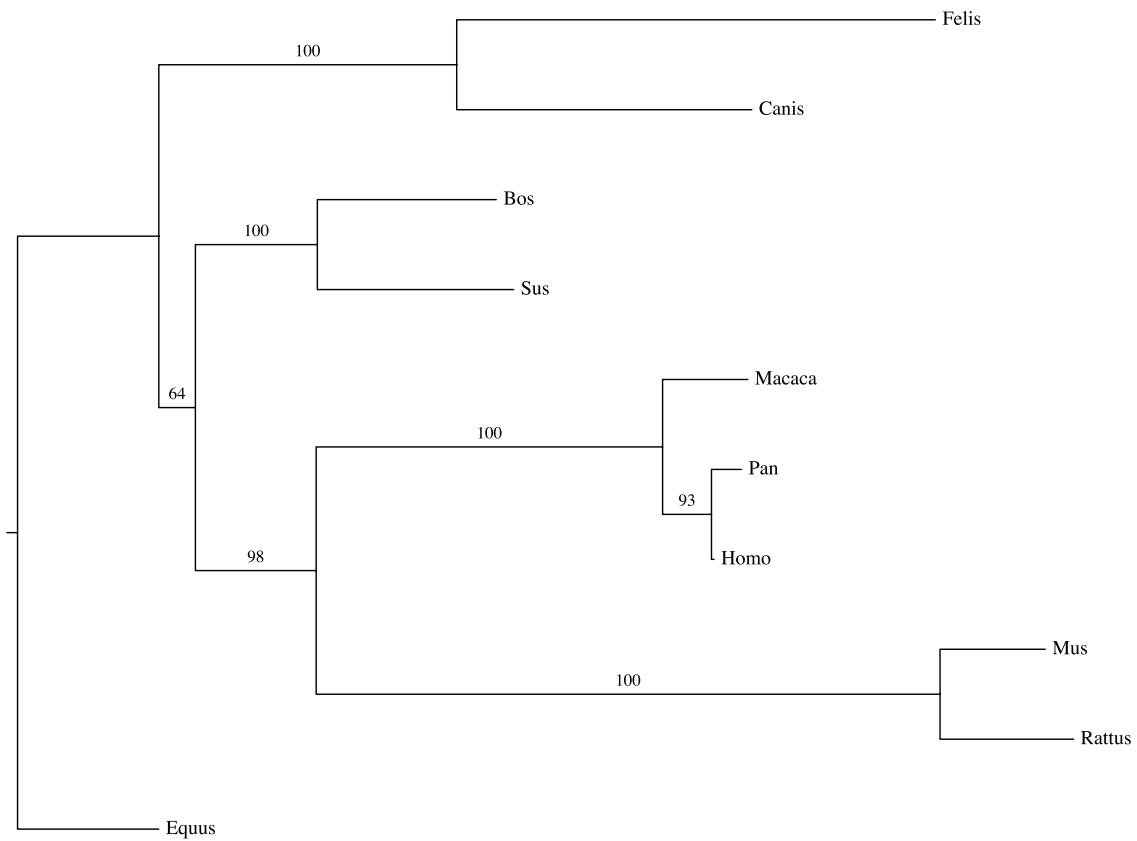

Supplement: Additional file 5 — Supplementary Figure 2: Maximum likelihood phylogeny of mammalian CCR2 gene sequences. A phylogeny of mammalian CCR2 gene sequences. The tree was produced using PHYML with the GTR nucleotide model, a discrete gamma model with four categories, and a shape parameter of 0.5867. Bootstrapping was performed with 100 replicates. Bootstrap support is indicated at nodes. [file 1471-2148-10-139-S5.PDF]

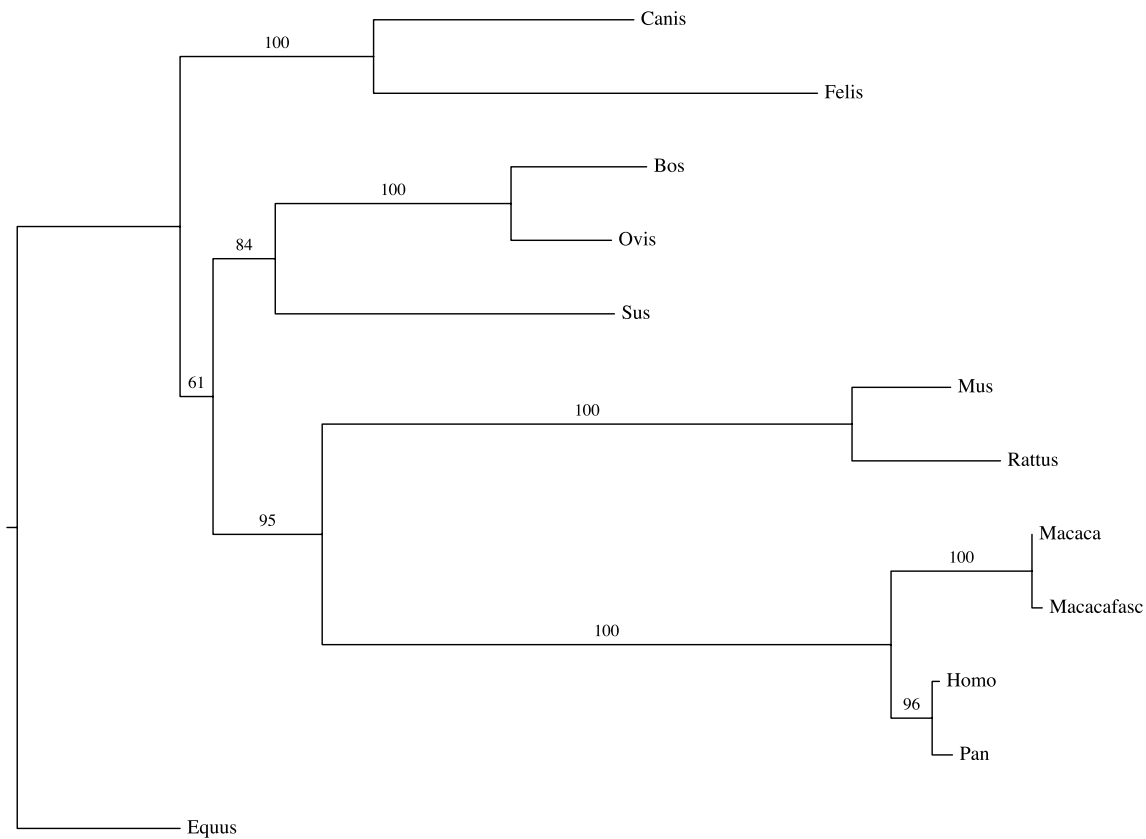

Supplement: Additional file 6 — Supplementary Figure 3: Maximum likelihood phylogeny of mammalian CCR3 gene sequences. A phylogeny of mammalian CCR3 gene sequences. The tree was produced using PHYML with the GTR nucleotide model, a discrete gamma model with four categories, proportion of invariable sites 0.3247 and a shape parameter of 2.8971. Bootstrapping was performed with 100 replicates. Bootstrap support is indicated at nodes. [file 1471-2148-10-139-S6.PDF]

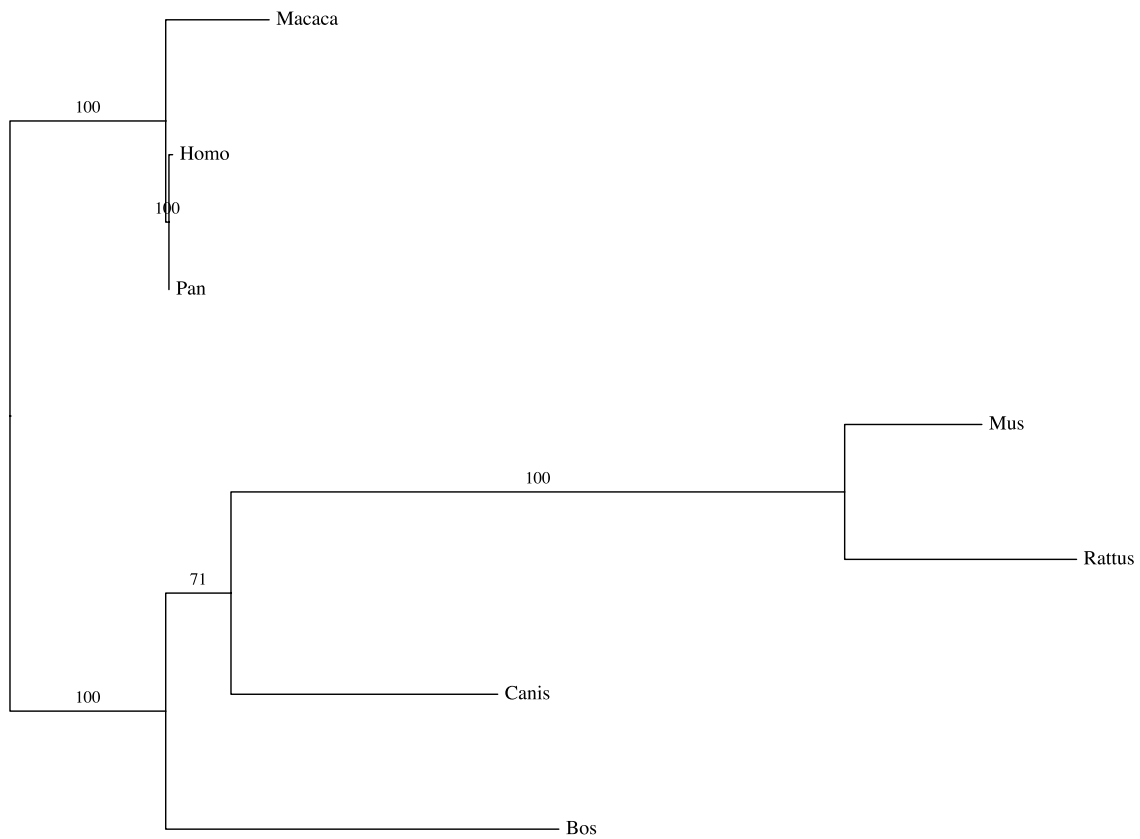

Supplement: Additional file 7 — Supplementary Figure 4: Maximum likelihood phylogeny of mammalian CCR4 gene sequences. A phylogeny of mammalian CCR4 gene sequences. The tree was produced using PHYML with the GTR nucleotide model, a discrete gamma model with four categories, proportion of invariable sites 0.5901 and an estimated shape parameter of 33.284. Bootstrapping was performed with 100 replicates. Bootstrap support is indicated at nodes. [file 1471-2148-10-139-S7.PDF]

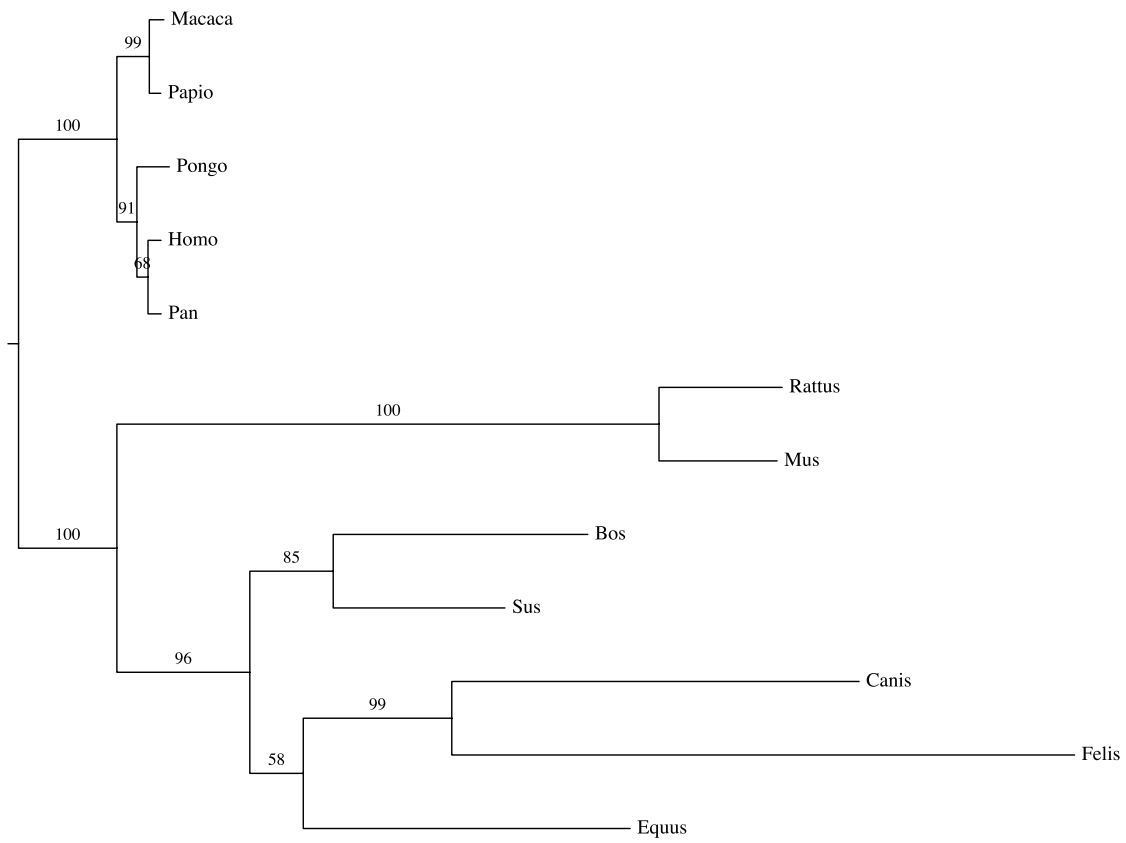

Supplement: Additional file 8 — Supplementary Figure 5: Maximum likelihood phylogeny of mammalian CCR5 gene sequences. A phylogeny of mammalian CCR5 gene sequences. The tree was produced using PHYML with the HKY nucleotide model, a discrete gamma model with four categories, and a shape parameter of 0.451. Bootstrapping was performed with 100 replicates. Bootstrap support is indicated at nodes. [file 1471-2148-10-139-S8.PDF]

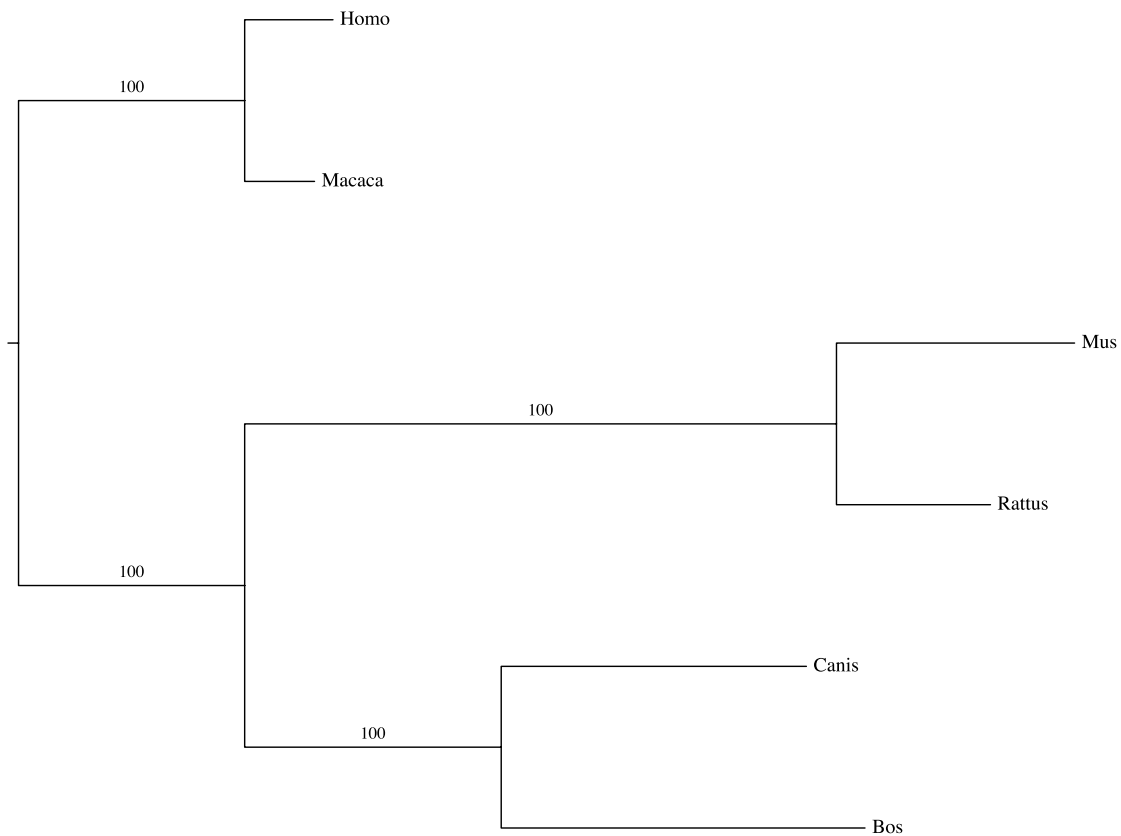

Supplement: Additional file 9 — Supplementary Figure 6: Maximum likelihood phylogeny of mammalian CCR6 gene sequences. A phylogeny of mammalian CCR6 gene sequences. The tree was produced using PHYML with the JC69 nucleotide model, a discrete gamma model with four categories, proportion of invariable sites 0.4667, and an estimated shape parameter of 100. Bootstrapping was performed with 100 replicates. Bootstrap support is indicated at nodes. [file 1471-2148-10-139-S9.PDF]

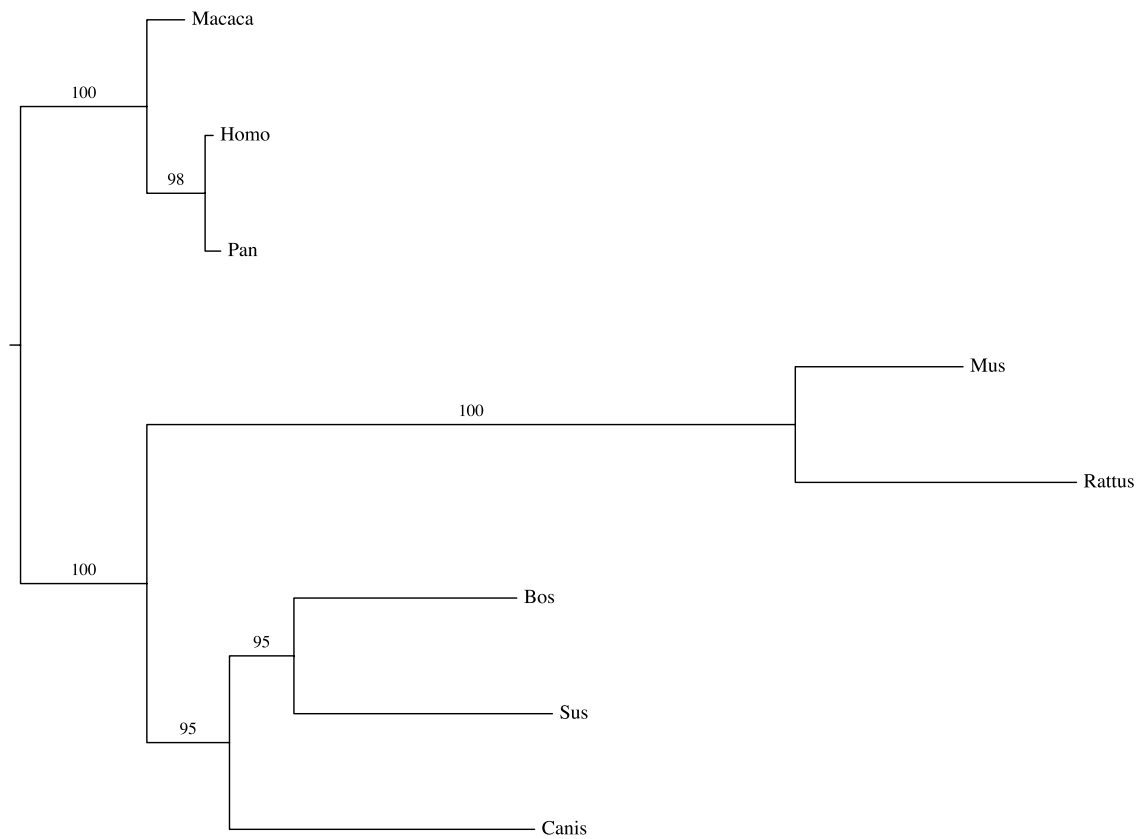

Supplement: Additional file 10 — Supplementary Figure 7: Maximum likelihood phylogeny of mammalian CCR7 gene sequences. A phylogeny of mammalian CCR7 gene sequences. The tree was produced using PHYML with the GTR nucleotide model, a discrete gamma model with four categories, and a shape parameter of 0.257. Bootstrapping was performed with 100 replicates. Bootstrap support is indicated at nodes. [file 1471-2148-10-139-S10.PDF]

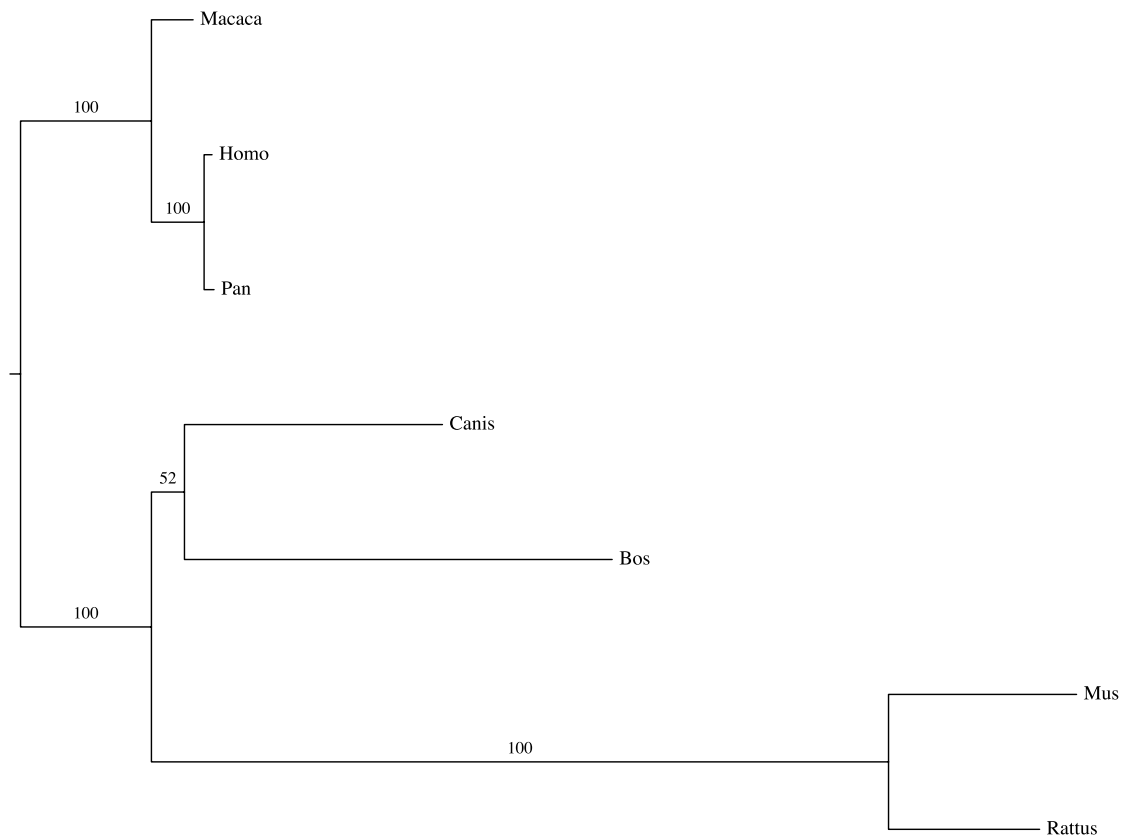

Supplement: Additional file 11 — Supplementary Figure 8: Maximum likelihood phylogeny of mammalian CCR8 gene sequences. A phylogeny of mammalian CCR8 gene sequences. The tree was produced using PHYML with the HKY nucleotide model, a discrete gamma model with four categories, and a shape parameter of 0.6482. Bootstrapping was performed with 100 replicates. Bootstrap support is indicated at nodes. [file 1471-2148-10-139-S11.PDF]

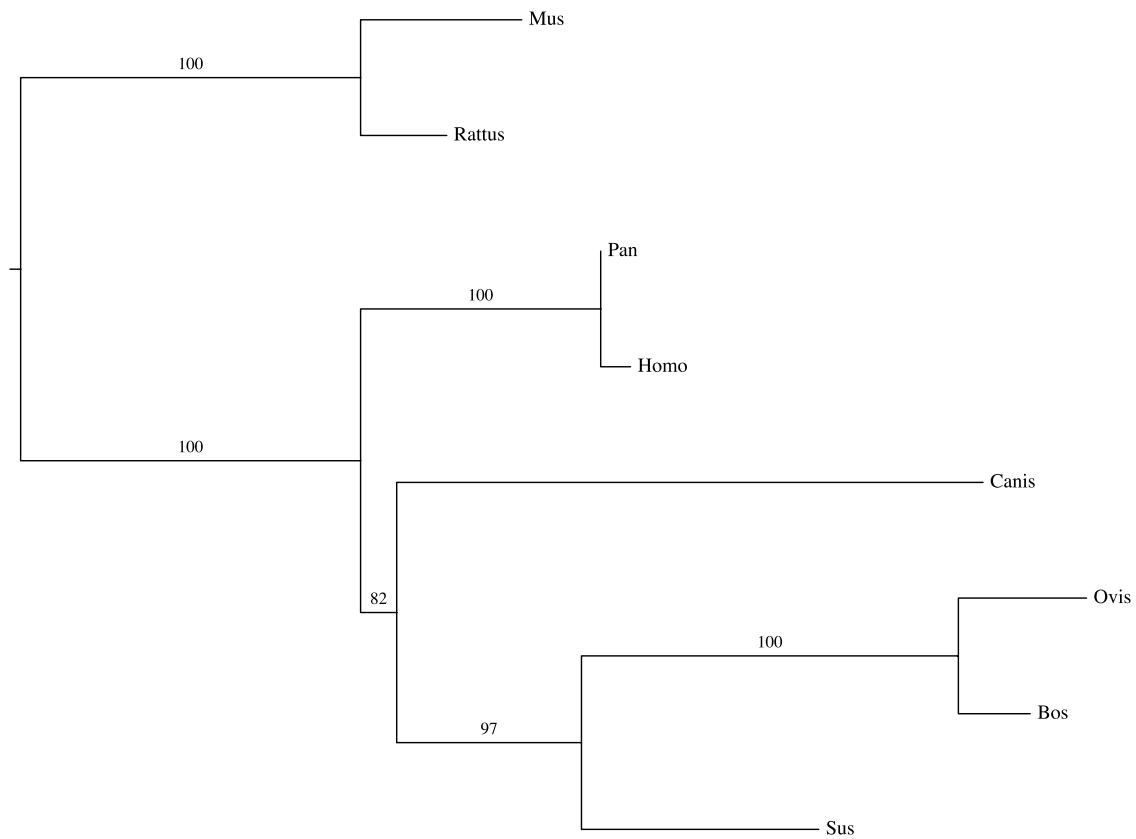

Supplement: Additional file 12 — Supplementary Figure 9: Maximum likelihood phylogeny of mammalian CCR9 gene sequences. A phylogeny of mammalian CCR9 gene sequences. The tree was produced using PHYML with the HKY nucleotide model, a discrete gamma model with four categories, and a shape parameter of 0.3547. Bootstrapping was performed with 100 replicates. Bootstrap support is indicated at nodes. [file 1471-2148-10-139-S12.PDF]

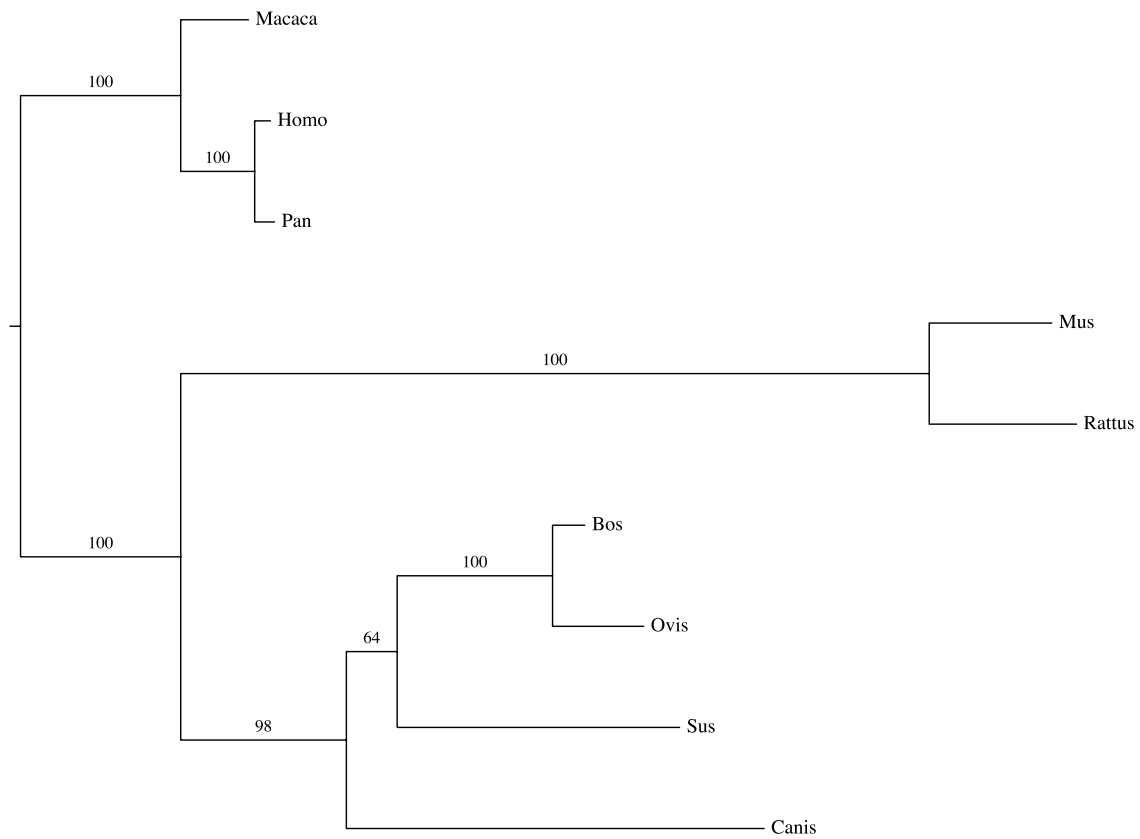

Supplement: Additional file 13 — Supplementary Figure 10: Maximum likelihood phylogeny of mammalian CCR10 gene sequences. A phylogeny of mammalian CCR10 gene sequences. The tree was produced using PHYML with the GTR nucleotide model, a discrete gamma model with four categories, and a shape parameter of 0.2439. Bootstrapping was performed with 100 replicates. Bootstrap support is indicated at nodes. [file 1471-2148-10-139-S13.PDF]
